# Inhibiting Neddylation with MLN4924 Suppresses Growth and Delays Multicellular Development in *Dictyostelium discoideum*

**DOI:** 10.3390/biom11030482

**Published:** 2021-03-23

**Authors:** Robert J. Huber, William D. Kim, Sabateeshan Mathavarajah

**Affiliations:** 1 Department of Biology, Trent University, Peterborough, ON K9L 0G2, Canada; 2 Environmental and Life Sciences Graduate Program, Trent University, Peterborough, ON K9L 0G2, Canada; williamkim@trentu.ca; 3 Department of Pathology, Dalhousie University, Halifax, NS B3H 4R2, Canada; smathavarajah@dal.ca

**Keywords:** neddylation, NEDD8, *Dictyostelium discoideum*, social amoeba, MLN4924, post-translational modification, growth, chemotaxis, development

## Abstract

Neddylation is a post-translational modification that is essential for a variety of cellular processes and is linked to many human diseases including cancer, neurodegeneration, and autoimmune disorders. Neddylation involves the conjugation of the ubiquitin-like modifier neural precursor cell expressed developmentally downregulated protein 8 (NEDD8) to target proteins, and has been studied extensively in various eukaryotes including fungi, plants, and metazoans. Here, we examine the biological processes influenced by neddylation in the social amoeba, *Dictyostelium discoideum*, using a well-established inhibitor of neddylation, MLN4924 (pevonedistat). NEDD8, and the target of MLN4924 inhibition, NEDD8-activating enzyme E1 (NAE1), are highly conserved in *D. discoideum* (Nedd8 and Nae1, respectively). Treatment of *D. discoideum* cells with MLN4924 increased the amount of free Nedd8, suggesting that MLN4924 inhibited neddylation. During growth, MLN4924 suppressed cell proliferation and folic acid-mediated chemotaxis. During multicellular development, MLN4924 inhibited cyclic adenosine monophosphate (cAMP)-mediated chemotaxis, delayed aggregation, and suppressed fruiting body formation. Together, these findings indicate that neddylation plays an important role in regulating cellular and developmental events during the *D. discoideum* life cycle and that this organism can be used as a model system to better understand the essential roles of neddylation in eukaryotes, and consequently, its involvement in human disease.

## 1. Introduction

Ubiquitin, or ubiquitin-like modifiers such as neural precursor cell expressed developmentally downregulated protein 8 (NEDD8), target both proteins and lipids to control their degradation, subcellular localization, macromolecular interactions, and enzymatic activity [1,2]. NEDD8 is conjugated to a target protein at a C-terminal lysine through a process known as neddylation. Neddylation was first observed in the *Saccharomycescerevisiae* Skp1–Cullin–F-box (SCF) complex, where cullin-1 was found conjugated to Rub1 (ortholog of NEDD8) [3,4]. Cullin proteins, which are components of the cullin–RING ligase (CRL) superfamily (the SCF complex is a part of this superfamily), are one of the main targets of neddylation in eukaryotic cells [5]. They serve as scaffolds for multi-subunit ubiquitin ligases that target proteins for ubiquitination [6]. The assembly of CRL complexes is modulated by cullin-associated and neddylation dissociated 1 (CAND1), which binds and sequesters cullin proteins to prevent CRL assembly [7,8]. The neddylation of cullin proteins causes the displacement of CAND1, thus facilitating CRL assembly [9]. While cullin proteins are the most well-studied targets of neddylation, non-cullin targets of neddylation have also been reported in a variety of organisms, including *Schizosaccharomyces pombe* and *Arabidopsis thaliana* [10,11,12].

Cycles of neddylation and de-neddylation work in tandem to facilitate the downstream regulation of target substrates (e.g., transcription factors, DNA repair proteins, growth factor receptors, etc.) in a dynamic and essential manner [13,14,15,16,17]. Consequently, abnormalities in neddylation are associated with a variety of human diseases including cancer, neurodegeneration, and autoimmune disorders [18,19,20]. In addition, the complete loss of neddylation is lethal in many eukaryotic organisms [4,21,22]. Although neddylation has been well-studied in a variety of organisms including parasitic protozoa, fungi, plants, animals, and humans, the pathway has not yet been explored in detail in amoebozoa such as *Dictyostelium discoideum* [23,24,25,26,27]. *D. discoideum* is a unikont that emerged at least 600 million years ago, prior to the fungi–animal split [28]. The life cycle of *D. discoideum* is comprised of both unicellular and multicellular phases [29]. Thus, it can be used to gather insight into how neddylation coordinates both unicellular (e.g., cell proliferation) and multicellular (e.g., tissue remodeling) processes in more complex eukaryotes, all in one organism. Once amoebae are starved, they aggregate via chemotaxis towards cyclic adenosine monophosphate (cAMP) to form a multicellular mound (0–10 h). Following mound formation, cells differentiate into either pre-stalk (~20% of population) or pre-spore (~80% of population) cells. These cell types then progress through the different developmental stages of the life cycle including the tipped mound (12–14 h), motile pseudoplasmodium (or slug) (16–18 h), culminant (20–22 h), and eventually, the fruiting body (24 h).

The *D. discoideum* genome encodes five proteins that contain a cullin homology domain (CulA-E encoded by *culA-E*) [30]. In terms of similarity, CulA is most similar to CUL1, CulB to CUL1 and CUL2, CulC to CUL3, CulD to CUL4A and CUL4B, and CulE to CUL1, CUL2, CUL5 (Table S1). Previous work showed that FLAG-tagged CulE is neddylated in slug-stage cells and that it interacts with orthologs of the mammalian neddylation machinery [31]. De-neddylation through the constitutive photomorphogenesis 9 (COP9) signalosome (CSN) has also been studied in *D. discoideum* and was revealed to play an important role in regulating cell proliferation [32]. The CSN is conserved across eukaryotes, from yeast to humans, and loss of CSN subunits results in aberrant phenotypes in all organisms studied to date [15,33,34,35,36,37,38,39,40,41,42]. Intriguingly, *D. discoideum* was identified as one of the earliest eukaryotes with a genome encoding an intact eight-subunit CSN [43]. These findings, coupled with the presence of an ortholog of human NEDD8 in *D. discoideum* (Nedd8), suggest that the neddylation pathway is conserved in *D. discoideum*.

In this study, we examine the role of the neddylation during the *D. discoideum* life cycle using the well-established neddylation inhibitor, MLN4924 (pevonedistat), which has been explored as its potential therapeutic for treating cancer, chronic liver diseases, and autoimmune diseases [15,44,45,46,47,48,49]. We identified previously unknown roles for neddylation in regulating *D. discoideum* growth, chemotaxis, aggregation, and fruiting body formation. We also establish *D. discoideum* as a model system for studying neddylation and highlight the potential of this organism to improve our understanding of the biological roles of neddylation and how this post-translational modification evolved in different eukaryotes. This work also identifies MLN4924 as an effective inhibitor for studying neddylation in *D. discoideum* and showcases the organism as a biomedical model system for informing work that evaluates the therapeutic potential of MLN4924.

## 2. Materials and Methods

### 2.1. Cells, Media, Buffers, Chemicals, and Antibodies

AX3 cells were obtained from the Dicty Stock Center [30]. Cells were grown and maintained on SM agar with *Klebsiella aerogenes* as well as axenically in HL5 medium at room temperature and 150 rpm [50]. Cultures were supplemented with 100 μg/mL ampicillin and 300 μg/mL streptomycin sulfate to prevent bacterial growth. HL5 medium was purchased from Formedium (Hunstanton, Norfolk, United Kingdom). KK2 buffer was composed of 0.7 g/L K_2_HPO_4_ and 2.2 g/L KH_2_PO_4_, pH 6.5. MLN4924 (pevonedistat) (catalog number: 505477), folic acid (catalog number: F7876), and cAMP (catalog number: A9501) were purchased from Sigma-Aldrich Canada Corporation (Oakville, ON, Canada). MLN4924 was dissolved in dimethyl sulfoxide (DMSO), which served as the control treatment in all experiments. Rabbit monoclonal anti-NEDD8 (19E3) (catalog number: 2754), goat anti-rabbit IgG HRP (catalog number: 7074), and horse anti-mouse IgG HRP (catalog number: 7076) were purchased from New England Biolabs Limited (Whitby, ON, Canada). Mouse monoclonal anti-β-actin (C4) (catalog number: sc-47778) was purchased from Santa Cruz Biotechnology Incorporated (Dallas, TX, USA). Rabbit polyclonal anti-CmfA was provided as a gift by Dr. Richard Gomer [51]. Rabbit polyclonal anti-CadA was generated and validated in a previous study [52]. Mouse monoclonal anti-α-actinin (catalog number: 47-18-9) was purchased from the Developmental Studies Hybridoma Bank (University of Iowa, Iowa City, IA, USA) [53].

### 2.2. Cell Proliferation Assay

Cell proliferation was assessed as previously described [54]. Briefly, cells in the mid-log phase of growth (1–5 × 106 cells/mL) were diluted to 2 × 10^5^ cells/mL in fresh HL5 medium ± MLN4924 (50 µM, 100 µM) and incubated at room temperature and 150 rpm. Cell concentrations were measured every 24 h over a 120-h period using a hemocytometer. Statistical significance was assessed in GraphPad Prism 8 using two-way ANOVA followed by Bonferroni post-hoc analysis (GraphPad Software Incorporated, La Jolla, CA, USA). A *p*-value < 0.05 was considered significant and *n* represents the number of biological replicates that were analyzed.

### 2.3. Radial Bioassay of Chemotaxis

Chemotaxis towards folic acid and cAMP was assessed using a radial bioassay [55,56]. Briefly, cells were deposited into Petri dishes, submerged in HL5, and left to grow overnight. The following day, confluent cells were harvested, washed twice with KK2 buffer, and then plated (0.25 µL of 1.5 × 10^8^ cells/mL suspension) on 0.5% agar/KK2 ± folic acid (50 µM) or cAMP (10 µM). MLN4924 (50 µM) was added to dishes containing folic acid or cAMP to assess the effect of MLN4924 on folic acid-mediated and cAMP-mediated chemotaxis. Cell spots were imaged at 0 and 6 h using a Nikon Ts2R-FL inverted microscope equipped with a Nikon Digital Sight Qi2 monochrome camera (Nikon Canada Incorporated Instruments Division, Mississauga, Ontario, Canada). Images were viewed using NIS Elements Basic Research and analyzed using Fiji/ImageJ. The amount of migration was determined by measuring the diameters of cell spots at the 0-h time point and subtracting those values from the diameters of cell spots after 6 h. Statistical significance was assessed in GraphPad Prism 8 using the one sample *t*-test. A *p*-value < 0.05 was considered significant and n represents the number of biological replicates that were analyzed.

### 2.4. Aggregation Assay

Aggregation was assessed as previously described [57]. Briefly, cells were deposited into 6-well dishes, submerged in HL5, and left to grow overnight. The following day, confluent cells were washed twice with KK2 buffer and then submerged in KK2 buffer ± MLN4924 (1 µM, 10 µM, 50 µM). Cells were imaged using a Nikon Ts2R-FL inverted microscope equipped with a Nikon Digital Sight Qi2 monochrome camera. Images were viewed using NIS Elements Basic Research. To determine the effect of MLN4924 on the amounts of free Nedd8, CmfA, and CadA, cells were deposited into Petri dishes, submerged in HL5, and left to grow overnight. The following day, confluent cells were starved in KK2 buffer ± MLN4924 (50 µM) for 6 h, after which time the conditioned buffer was harvested and cells were lysed with a buffer containing 0.5% NP-40, 150 mM NaCl, 50 mM Tris, pH 7.5, and a protease inhibitor tablet (i.e., NP-40 lysis buffer) (Fisher Scientific Company, Ottawa, Ontario, Canada). Samples were stored at −80 °C for future use.

### 2.5. Multicellular Development Assay

To determine the amount of free Nedd8 in cells during the different stages of the *D. discoideum* life cycle, cells were deposited into Petri dishes, submerged in HL5, and left to grow overnight. The following day, confluent growth-phase cells were harvested and lysed with NP-40 lysis buffer. Samples were stored at −80 °C for future use. Confluent cells were also harvested, washed twice with KK2 buffer, and deposited (5 × 10^6^ total cells) on 0.5% agar/KK2 contained within 100 mm × 60 mm Petri dishes. The Petri dishes were placed on top of a moist paper towel and then covered with plastic wrap and aluminum foil to maintain a humid environment and ensure synchronous development. Cells were harvested every 4 h over a 24-h period and then lysed with NP-40 lysis buffer. All samples were stored at −80 °C for future use. To determine the effect of MLN4924 on multicellular development, cells were deposited into Petri dishes, submerged in HL5, and left to grow overnight. The following day, confluent cells were harvested and washed twice with KK2 buffer. Cells were then deposited (25 µL of 2 × 10^7^ cells/mL suspension) onto 0.5% agar/KK2 ± MLN4924 (1 µM, 10 µM, 50 µM) contained within 60 mm × 15 mm Petri dishes. All development dishes were imaged using a Leica EZ4W stereomicroscope equipped with an internal 5MP CMOS camera (Leica Microsystems Incorporated, Concord, Ontario, Canada). The statistical significance for the effect of MLN4924 on multicellular development was assessed in GraphPad Prism 8 using one-way ANOVA followed by Tukey’s multiple comparison’s test. A *p*-value < 0.05 was considered significant and *n* represents the number of biological replicates that were analyzed.

### 2.6. SDS-PAGE and Western Blotting

Protein quantification was performed using a Qubit 2.0 Fluorometer (Fisher Scientific Company, Ottawa, ON, Canada). Whole cell lysates and samples of conditioned buffer were resuspended in Laemmli sample buffer [58]. Samples were then heated at 95 °C for 5 min. SDS-PAGE and Western blotting were performed using standard methods (2-h incubation at room temperature for primary and secondary antibodies in 5% milk/TBST), except incubations with anti-NEDD8, which were performed overnight at 4 °C with rotation. The following primary and secondary antibodies were used: anti-NEDD8 (1:1000), anti-β-actin (1:2000), anti-CmfA (1:1000), anti-CadA (1:1000), anti-α-actinin (1:1000), anti-rabbit IgG HRP (1:4000), and anti-mouse IgG HRP (1:4000). Immunoblots were digitally scanned using the ChemiDoc MP Imaging System (Bio-Rad Laboratories Limited, Mississauga, Ontario, Canada). Protein bands were quantified using Fiji/ImageJ and standardized against the levels of β-actin or α-actinin. Statistical significance was assessed in GraphPad Prism 8 using the one-sample *t*-test. A *p*-value < 0.05 was considered significant and *n* represents the number of biological replicates that were analyzed.

### 2.7. Bioinformatics

Amino acid alignments were performed using the MUSCLE alignment tool available on MEGAX [59]. The alignment display was generated using the ESPRIPT 3.0 tool [60]. A structural homology model of *D. discoideum* Nae1 was generated using Phyre2 and the 3DBR crystal structure template from the Research Collaboratory for Structural Bioinformatics Protein Data Bank [61,62].

## 3. Results

### 3.1. The Putative Neddylation Pathway in D. discoideum Resembles the Metazoan Neddylation Pathway

**Figure 1:**
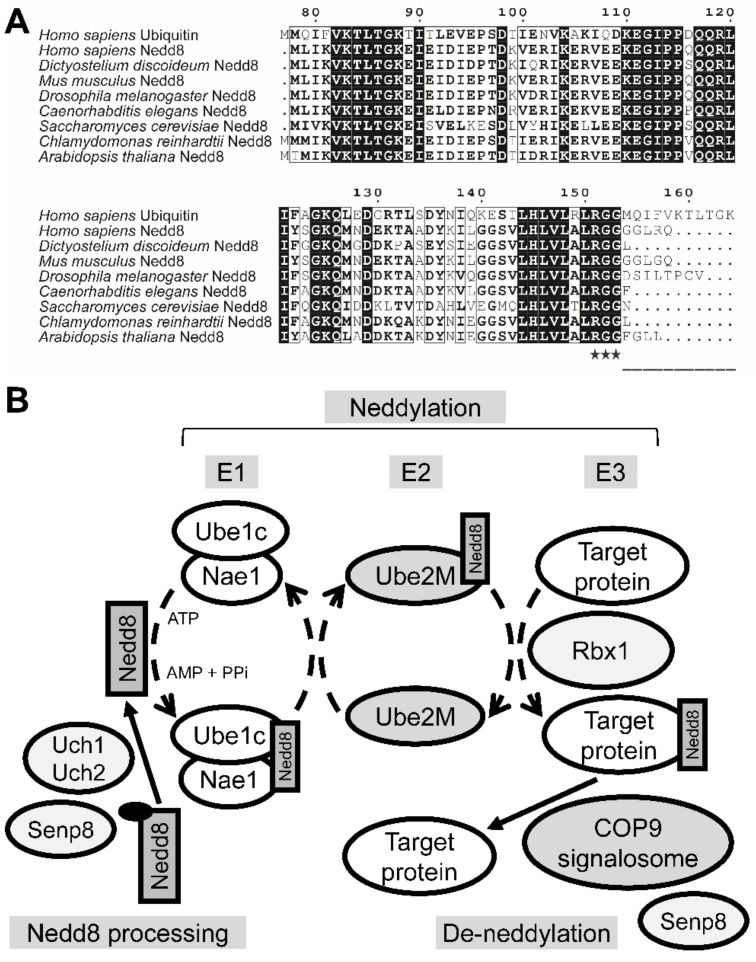
The putative neddylation pathway in *Dictyostelium discoideum.* (**A**) The *D. discoideum* neural precursor cell expressed developmentally downregulated protein 8 (Nedd8) is highly conserved and contains a single residue C-terminal extension. Alignments between human polyubiquitin C (P0CG48, Uniprot), human NEDD8 (Q15843, Uniprot), and NEDD8 orthologs in representative species (*Mus musculus*, P29595, Uniprot; *Drosophila melanogaster*, Q9VJ33, Uniprot; *Caenorhabditis elegans*, Q93725, Uniprot; *Saccharomyces cerevisiae*, Q03919, Uniprot; *Dictyostelium discoideum*, Q54XV3, Uniprot; *Chlamydomonas reinhardtii*, A8IZZ4, Uniprot; and *Arabidopsis thaliana*, Q9SHE7, Uniprot) were compiled and aligned using the MUSCLE alignment tool available on MEGAX. The sequence position number corresponds to the region of human ubiquitin that aligns to NEDD8. The RGG motif for NEDD8 conjugation is marked by stars. The C-terminal extension for NEDD8 and orthologous sequences is underlined. Sequence similarity is depicted in terms of % equivalent for the colouring scheme. The alignment display was generated using the ESPRIPT 3.0 tool. (**B**) The proposed model showing how Nedd8 is conjugated to target proteins in *D. discoideum*. Nedd8 is translated as an inactive protein with a C-terminal extension (black oval) that is cleaved by ubiquitin C-terminal hydrolase 1 and 2 (Uch1 and Uch2, respectively) and sentrin-specific protease 8 (Senp8) to generate the mature Nedd8 protein, which is then transferred through an E1–E2–E3-like cascade to a target protein via ubiquitin-activating enzyme E1C (Ube1C), Nedd8-activating enzyme E1 (Nae1), ubiquitin-conjugating enzyme E2 M (Ube2M), and the E3 ubiquitin protein ligase Rbx1. De-neddylation is carried out by the constitutive photomorphogenesis 9 (COP9) signalosome. Senp8 also contributes to de-neddylation but is not essential to the process.

NEDD8 is the ubiquitin-like modifier that most closely resembles ubiquitin. Like ubiquitination, the conjugation of NEDD8 to target proteins (e.g., cullin proteins) follows an E1–E2–E3-like cascade [63]. *D. discoideum* Nedd8 (DDB0238041) is a 77-amino-acid protein that shares 82% exact identity (63/76 amino acids) and 92% positive similarity (70/76 amino acids) with the 81-amino-acid human NEDD8 protein (Q15843, Uniprot) (Figure 1A). *D. discoideum* Nedd8 also shares 55% exact identity (43/77 amino acids) and 77% positive similarity (60/77 amino acids) with human polyubiquitin C (P0CG48, Uniprot) (Figure 1A). Akin to ubiquitin, NEDD8 is proteolytically processed to generate its mature form [64]. Once NEDD8 is translated into an inactive precursor, its short C-terminal amino acid extension is cleaved downstream of the RGG residues (Figure 1A). This C-terminal extension is only one-amino-acid long for *D. discoideum* Nedd8. In that sense, *D. discoideum* Nedd8 most closely resembles Nedd8 from *Chlamydomonas reinhardtii*, *Saccharomyces cerevisae*, and *Caenorhabditis elegans*.

Previous work on *D. discoideum* identified the proteins that interact with CulE, which included several orthologs of the mammalian neddylation machinery including the E3 ubiquitin protein ligase RBX1, CAND1, and subunits of the CSN [31]. The CSN, which is involved in de-neddylation, has also been characterized [32]. Using this information, we generated a putative model for neddylation/de-neddylation in *D. discoideum* (Figure 1B). The putative pathway includes orthologs of mammalian E1 (ubiquitin-activating enzyme E1C, Ube1C, DDB0238040; Nedd8-activating enzyme E1, Nae1, DDB0237981), E2 (ubiquitin-conjugating enzyme E2 M, Ube2M, DDB0238042), and E3 (Rbx1, DDB0231276), as well as the NEDD8-processing enzymes ubiquitin carboxyl-terminal hydrolase isozyme L1 (UCHL1) (ubiquitin C-terminal hydrolase 1, Uch1, DDB0304593), ubiquitin carboxyl-terminal hydrolase isozyme L5 (UCHL5) (ubiquitin C-terminal hydrolase 2, Uch2, DDB0233072), and sentrin-specific protease 8 (SENP8) (Senp8, DDB0304995) (Table S2). *D. discoideum* Uch1 also shows similarity to human ubiquitin carboxyl-terminal hydrolase isozyme L3 (UCHL3) (Table S2). However, unlike the mammalian pathway that utilizes two E2 proteins (UBE2M and UBE2F), the *D. discoideum* genome encodes a single E2 protein (Ube2M) that shares similarity to NEDD8-conjugating enzyme Ubc12 (UBE2M) (Table S2). The presence of NEDD8, NAE1, and cullin orthologs in *D. discoideum*, coupled with the neddylation of CulE, suggests that neddylation may play an important role during the *D. discoideum* life cycle.

**Figure 2:**
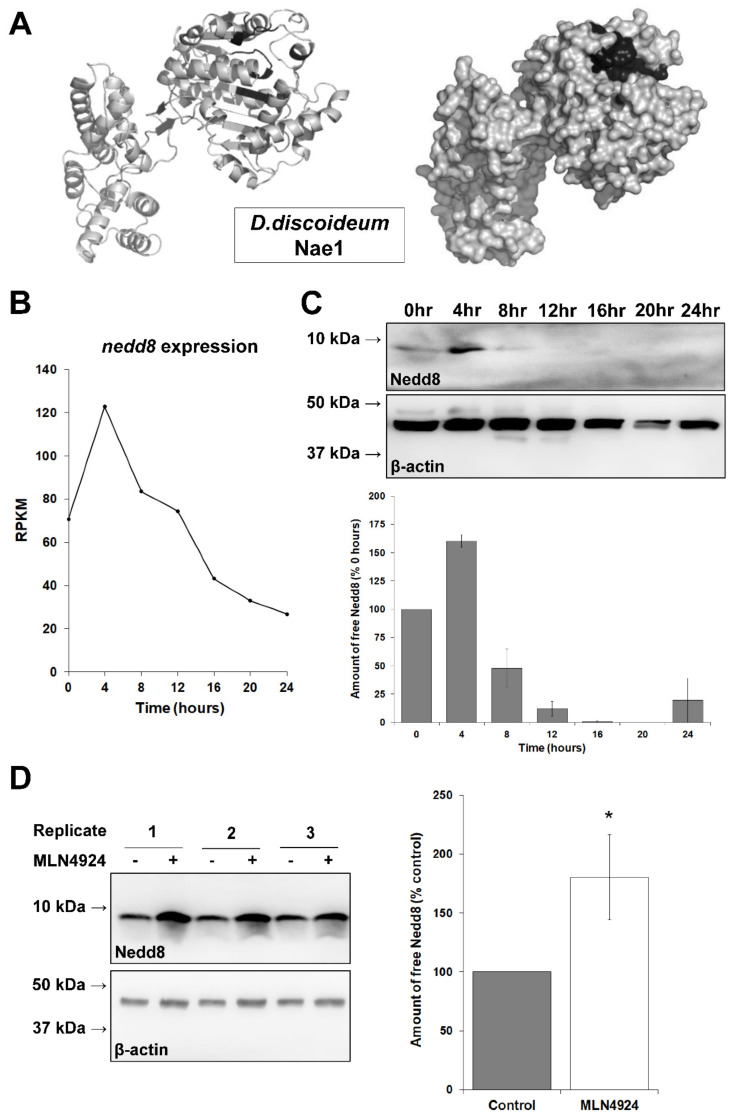
MLN4924 inhibits neddylation in *D. discoideum*. (**A**) Homology modeling of *D. discoideum* Nedd8-activating enzyme E1 (Nae1). The structure of Nae1 is highly conserved and predicted to bind MLN4924 (predicted binding pocket indicated in black). (**B**) Expression of *nedd8* during *D. discoideum* growth and multicellular development. The *x*-axis represents the time of development: 0 h (growth), 4 h (chemotaxis), 8 h (aggregate formation), 12 h (mound), 16 h (slug), 20 h (culminant), 24 h (fruiting body). Reads per kilobase of transcript per million mapped reads (RPKM). RNA-seq data were obtained from dictyExpress and re-plotted using Microsoft Excel. (**C**) Amount of free Nedd8 in cells during the different stages of *D. discoideum* development. Whole cell lysates (20 µg total protein) were separated by SDS-PAGE and analyzed by Western blotting with anti-NEDD8 and anti-β-actin (loading control). Western blots are representative of three biological replicates. Molecular weight markers (in kDa) are shown to the left of each blot. Nedd8 protein bands were quantified and standardized against the amount of β-actin. Data are presented as the mean amount of free Nedd8 (% 0 h) ± SEM (*n* = 3). (**D**) Effect of MLN4924 on the amount of free Nedd8. Cells were starved in KK2 buffer in the presence and absence of MLN4924 (50 µM, control: dimethyl sulfoxide) for 6 h, after which time cells were lysed. Whole cell lysates (50 µg total protein) were separated by SDS-PAGE and analyzed by Western blotting with anti-NEDD8 and anti-β-actin (loading control). Three representative biological replicates are shown. Molecular weight markers (in kDa) are shown to the left of each blot. Nedd8 protein bands were quantified and standardized against the amount of β-actin. Data are presented as the mean amount of free Nedd8 (% control) ± SEM (*n* = 19). Statistical significance was assessed using the one-sample *t*-test. * *p* < 0.05 vs. control.

MLN4924 is a synthetic drug that selectively inhibits NAE1 to prevent neddylation [44]. The inhibitor has been shown to bind NAE1 orthologs and inhibit neddylation in various organisms including yeast, zebrafish, and plants [65,66,67]. Homology modeling of *D. discoideum* Nae1 generated a structure that highly resembles human NAE1 (Phyre2 generated a structure with 100% confidence using human NAE1 as the template) (Figure 2A). At the amino acid level, the 520-amino-acid *D. discoideum* Nae1 protein shares 41% exact identity (219/524 amino acids) and 64% positive similarity (336/524 amino acids) with the 534-amino-acid human NAE1 protein (Q13564, Uniprot) (data not shown). Overall, these findings suggest that the neddylation pathway is conserved from *D. discoideum* to mammals.

### 3.2. MLN4924 Inhibits Neddylation in D. discoideum

The highly conserved structure of *D. discoideum* Nae1 suggests that MLN4924 has the capacity to bind Nae1 in *D. discoideum* and inhibit neddylation. To determine if MLN4924 inhibited neddylation in *D. discoideum*, we starved cells for 6 h in KK2 buffer in the presence and absence of MLN4924, since RNA-seq data show that *nedd8* expression peaks during the early stages of multicellular development and then falls to its lowest levels during terminal differentiation (Figure 2B) [68]. We used a highly specific antibody directed against human NEDD8 to confirm this trend at the protein level (Figure 2C). Following incubation with MLN4924, cells were lysed. Proteins were then separated by SDS-PAGE and analyzed by Western blotting using anti-NEDD8 (Figure 2D). The amount of free Nedd8 was 80 ± 36% higher in cells treated with MLN4924 compared to control cells, suggesting that neddylation was inhibited by MLN4924 (i.e., Nedd8 accumulated because it was not conjugated to target proteins). This finding indicated that MLN4924 could be used to study processes regulated by neddylation in *D. discoideum*.

### 3.3. MLN4924 Inhibits Cell Proliferation and Folic Acid-Mediated Chemotaxis in D. discoideum

**Figure 3:**
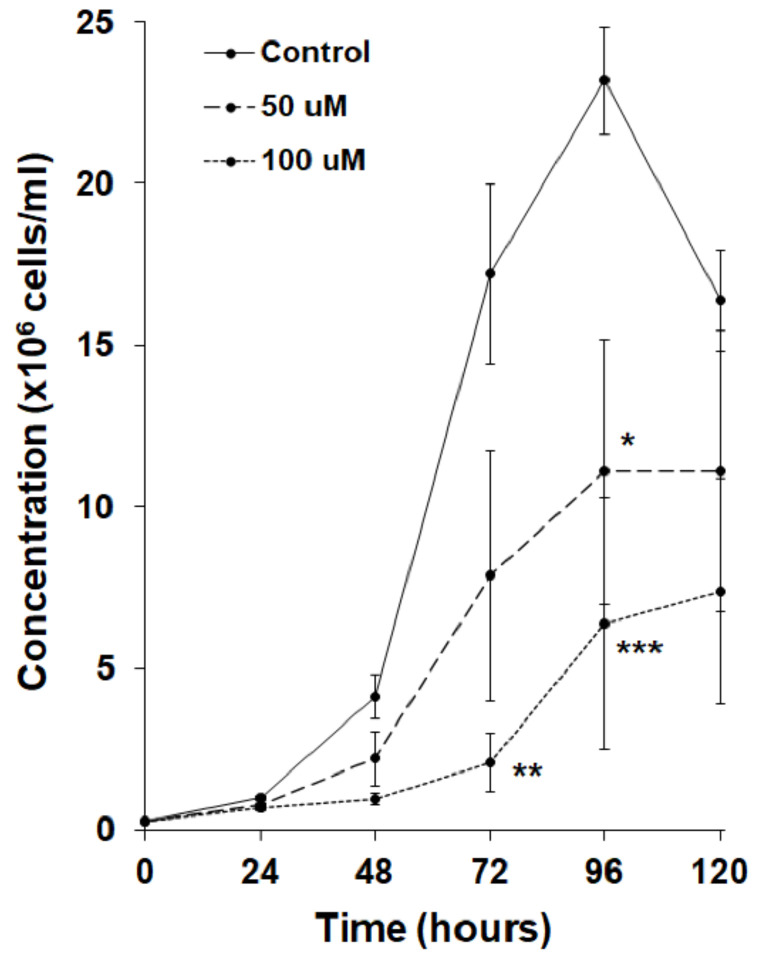
Effect of MLN4924 on cell proliferation. Cells were grown in HL5 medium in the presence and absence of MLN4924 (50 µM, 100 µM, control: dimethyl sulfoxide) over a period of 5 days (120 h). Data presented as the mean concentration (×10^6^ cells/mL) ± SEM (*n* ≥ 4). Statistical significance was assessed using two-way ANOVA followed by Bonferroni post-hoc analysis. Two-way ANOVA revealed a significant effect of MLN4924 treatment on the growth curves (*p* < 0.0001). * *p* < 0.05, ** *p* < 0.01, and *** *p* < 0.001 vs. control at the indicated time points as determined from Bonferroni post-hoc analysis.

**Figure 4:**
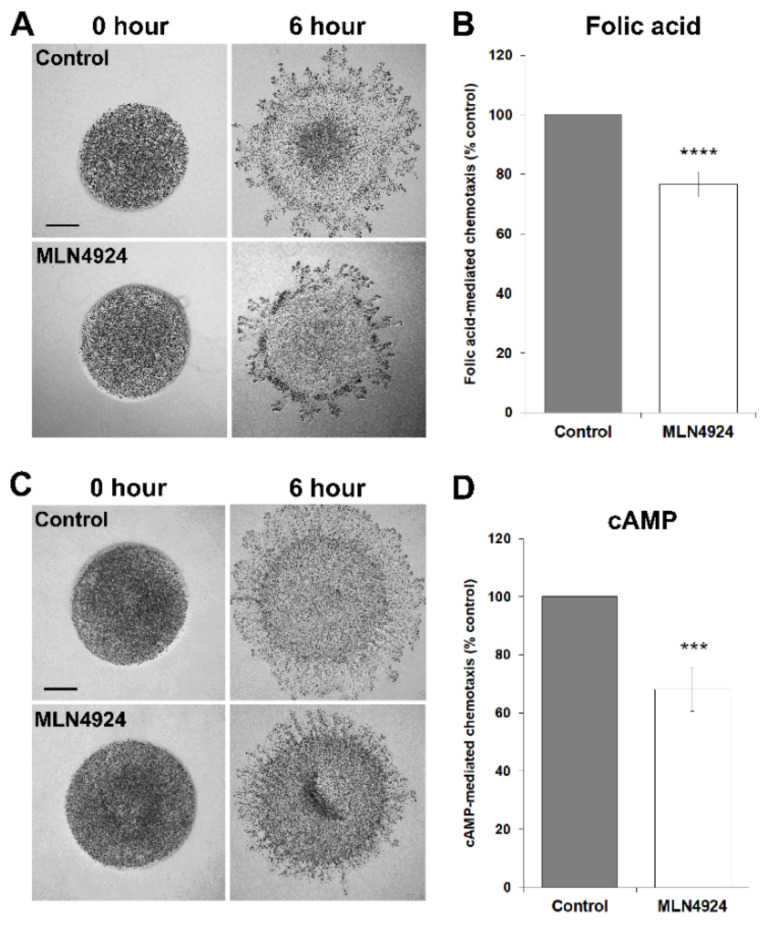
Effect of MLN4924 on folic acid-mediated and cyclic adenosine monophosphate (cAMP)-mediated chemotaxis. (**A**) Cells were deposited onto 0.5% agar/KK2/folic acid (50 µM) in the presence and absence of MLN4924 (50 µM, control: dimethyl sulfoxide). Images were taken once cells were deposited (0 h) and after 6 h. Scale bar = 500 μm. (**B**) The amount of migration towards folic acid was quantified as detailed in the Section 2. Data are presented as the mean folic acid-mediated chemotaxis (% control) ± SEM (*n* = 13). (**C**) Cells were deposited onto 0.5% agar/KK2/cAMP (10 µM) in the presence and absence of MLN4924 (50 µM, control: dimethyl sulfoxide). Images were taken once cells were deposited (0 h) and after 6 h. Scale bar = 500 μm. (**D**) The amount of migration towards cAMP was quantified as detailed in the Section 2. Data presented as mean cAMP-mediated chemotaxis (% control) ± SEM (*n* = 18). Statistical significance in (**B**) and (**D**) was assessed using the one-sample *t*-test. *** *p* < 0.001 and **** *p* < 0.0001 vs. control.

In humans, proteins are de-neddylated by the CSN5 subunit of the CSN [69]. Loss of CSN5 ortholog in *D. discoideum*, *csn5*, severely impairs cell proliferation, suggesting that the cycling of neddylation and de-neddylation is important during growth [32]. Based on these observations, we tested the effect of MLN4924 in cultures of axenically growing *D. discoideum* cells. MLN4924 significantly reduced cell proliferation in a dose-dependent manner (Figure 3). Since growth-phase cells locate their food source by chemotactically responding to the folic acid that is secreted by bacteria, we next examined the effect of MLN4924 on folic acid-mediated chemotaxis. For this experiment, we used the well-established radial bioassay that assesses the migration of a population of *D. discoideum* cells in response to a self-generated gradient of chemoattractant [55]. In this assay, MLN4924 inhibited the migration of *D. discoideum* cells in response to folic acid by 23 ± 4% (Figure 4A,B). Together, these findings suggest that neddylation plays a key role in regulating cell proliferation and folic acid-mediated chemotaxis during the growth-phase of the *D. discoideum* life cycle.

### 3.4. MLN4924 Treatment Inhibits cAMP-Mediated Chemotaxis and Delays the Aggregation of D. discoideum Cells

**Figure 5:**
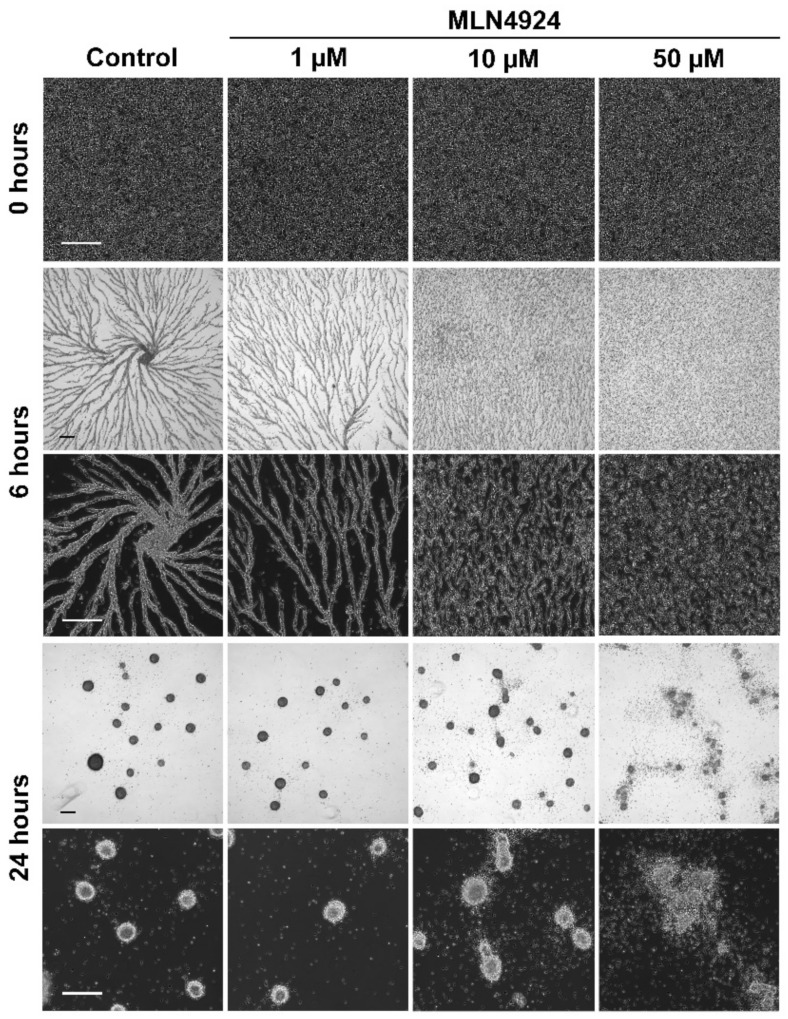
Effect of MLN4924 on aggregation. Cells were starved in KK2 buffer in the presence and absence of MLN4924 (1 µM, 10 µM, 50 µM, control: dimethyl sulfoxide). Cells were imaged at the indicated times. Scale bar = 250 µm. Images are representative of four independent experiments.

**Figure 6:**
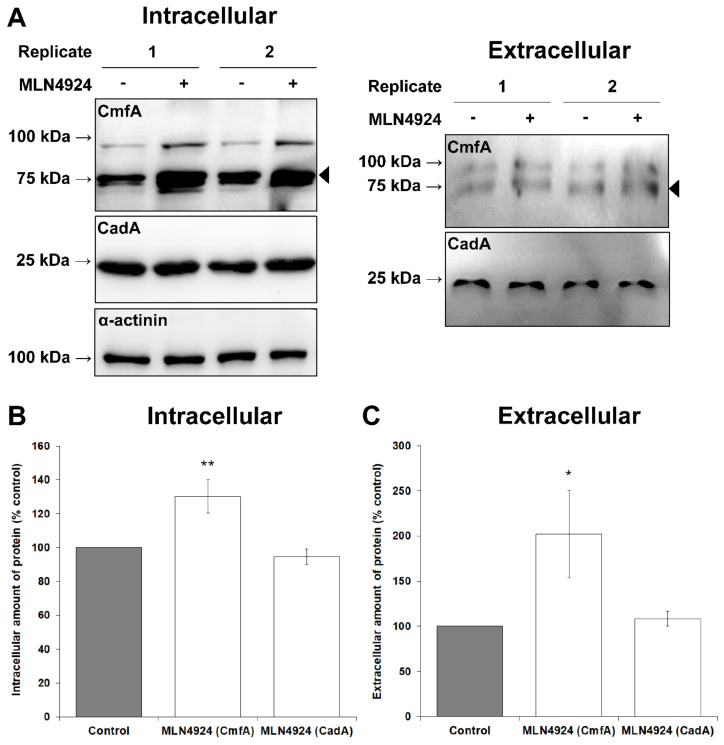
Effect of MLN4924 on the intracellular and extracellular amounts of conditioned media factor (CmfA) and calcium-dependent cell adhesion molecule A (CadA). (**A**) Cells were starved in KK2 buffer in the presence and absence of MLN4924 (50 µM, control: dimethyl sulfoxide) for 6 h, after which time the conditioned buffer was collected and cells were lysed. Whole cell lysates (WC, 50 µg total protein) and equal volumes of conditioned buffer (CB, 15 µL) were separated by SDS-PAGE and analyzed by Western blotting with anti-CmfA, anti-CadA, and anti-α-actinin (loading control). Two representative biological replicates are shown. Molecular weight markers (in kDa) are shown to the left of each blot. The bands corresponding to CmfA are indicated with a black arrow to the right of each blot. (**B**) CmfA and CadA protein bands in whole cell lysates were quantified and standardized against the amount of α-actinin. Data presented as mean intracellular amount of protein (% control) ± SEM (*n* = 32). (**C**) CmfA and CadA protein bands in samples of conditioned buffer were quantified. Data presented as mean extracellular amount of protein (% control) ± SEM (*n* = 12). Statistical significance in (**B**) and (**C**) was assessed using the one-sample *t*-test. * *p* < 0.05 and ** *p* < 0.01 vs. control.

CulA was previously shown to play a role in cAMP-mediated chemotaxis and cell aggregation [70]. Based on these observations, we assessed the effect of MLN4924 on cAMP-mediated chemotaxis, which drives multicellular aggregate formation during the early stages of multicellular development. In the radial bioassay, MLN4924 inhibited cAMP-mediated chemotaxis by 32 ± 8% compared to untreated cells (Figure 4C,D). When *D. discoideum* cells were starved in KK2 buffer with MLN4924, we observed a dose-dependent delay in aggregation (Figure 5). After 24 h, mounds formed by cells treated with 50 µM MLN4924 were amorphous in shape compared to control mounds with many cells not contributing to mound formation (Figure 5). To explore the mechanism underlying the delayed aggregation of MLN4924-treated cells, we examined the effect of MLN4924 on the intracellular and extracellular amounts of conditioned medium factor (CmfA) and calcium-dependent cell adhesion molecule A (CadA) after 6 h of starvation in KK2 buffer. CmfA is a ~75-kDa cell density-sensing secreted glycoprotein that enables starving cells to respond to pulses of cAMP [71]. CadA is a ~25-kDa protein that facilitates calcium-dependent cell-to-cell interactions during aggregation and development [72]. Treatment with MLN4924 increased the intracellular and extracellular amounts of CmfA by 30 ± 10% and 102 ± 48%, respectively, compared to untreated cells, suggesting that cells treated with MLN4924 attempt to compensate for the delayed aggregation by producing and secreting more CmfA (Figure 6). In contrast, there was no significant effect of MLN492 on the intracellular or extracellular amounts of CadA. Collectively, these results suggest an important role for neddylation in cAMP-mediated chemotaxis, aggregation, and mound formation during the early stages of *D. discoideum* development.

### 3.5. MLN4924 Treatment Inhibits Fruiting Body Formation during D. discoideum Development

**Figure 7:**
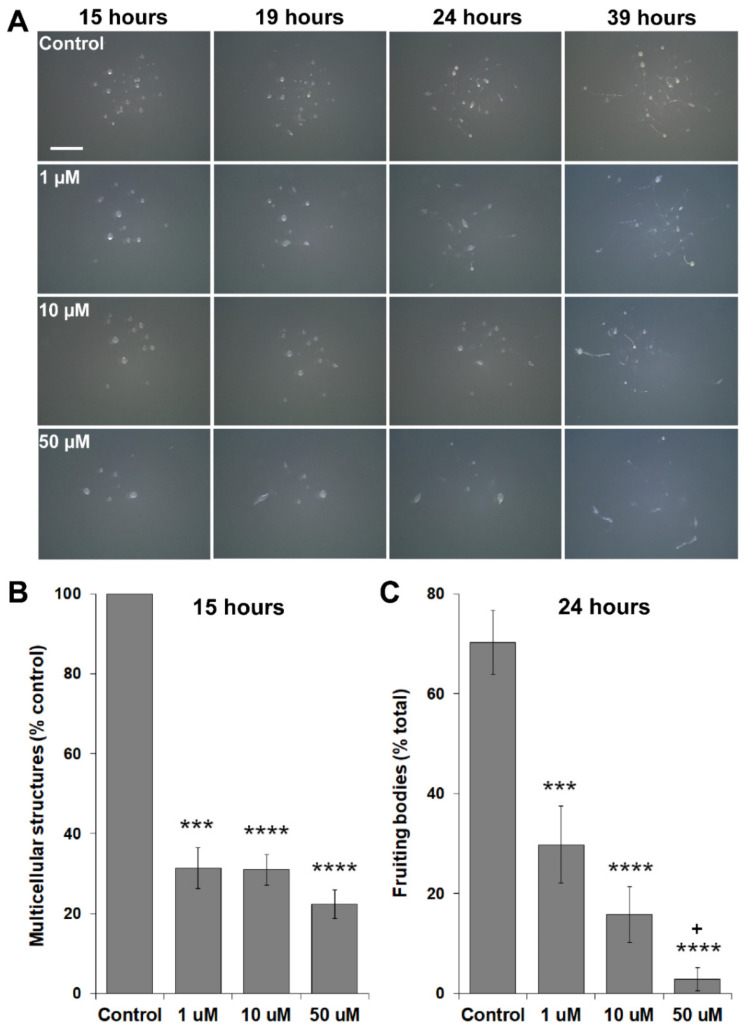
Effect of MLN4924 on multicellular development. (**A**) Cells were deposited onto 0.5% agar/KK2 in the presence and absence of MLN4924 (1 µM, 10 µM, 50 µM, control: dimethyl sulfoxide). Dishes were imaged at the indicated times. Scale bar = 0.25 cm. Images are representative of five independent experiments. (**B**) The number of multicellular structures observed after 15 h of development was quantified. Data are presented as the mean multicellular structures (% control) ± SEM (*n* ≥ 5). Statistical significance was assessed using the one-sample *t*-test. *** *p* < 0.001 and **** *p* < 0.0001 vs. control. (**C**) The percentage of fruiting bodies observed after 24 h of development was quantified. Data are presented as the mean fruiting bodies (% total) ± SEM (*n* ≥ 5). Statistical significance was assessed using one-way ANOVA followed by Tukey’s multiple comparison’s test. One-way ANOVA revealed a significant effect of MLN4924 treatment on fruiting body formation (*p* < 0.0001). *** *p* < 0.001 and **** *p* < 0.0001 vs. control as determined by Tukey’s multiple comparisons test. +*p* < 0.05 vs. 1 µM as determined by Tukey’s multiple comparisons test.

CulA and CulB have previously been shown to regulate morphogenesis and cell type differentiation during *D. discoideum* development [70,73]. In addition, *nae1*, the target of MLN4924 inhibition, is upregulated in spores [74]. For these reasons, we developed cells on agar to examine the potential role of neddylation in the later stages of multicellular development. MLN4924 treatment significantly reduced the formation of multicellular structures by ~70% and delayed the mid-to-late stages of development (e.g., pseudoplasmodium formation) (Figure 7A,B). After 24 h, MLN4924 dose-dependently inhibited fruiting body formation (Figure 7A,C). Development was severely impacted after treatment with 50 µM MLN4924. Under these conditions, only 3 ± 2% of multicellular structures developed into fruiting bodies after 24 h, compared to 70 ± 6% under control conditions (Figure 7A,C). Together, these findings suggest an important role for neddylation in the later stages of *D. discoideum* development.

## 4. Discussion

**Figure 8:**
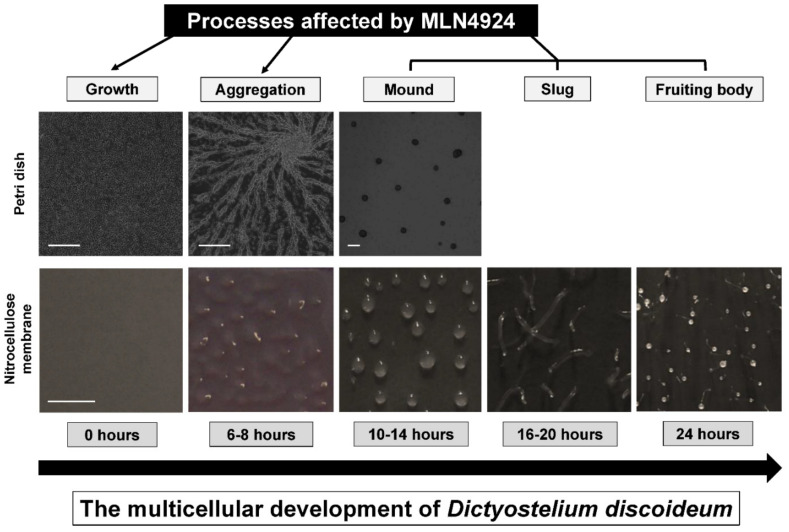
Processes affected by MLN4924 during the *D. discoideum* life cycle. MLN4924 inhibits cell proliferation, chemotaxis, aggregation, and fruiting body formation, indicating that neddylation/de-neddylation regulates these cellular and developmental events during the *D. discoideum* life cycle. Images show the development of *D. discoideum* cells in two experimental environments commonly used in the laboratory: (1) cells adhered to Petri dishes and submerged in KK2 buffer and (2) cells deposited onto nitrocellulose membranes soaked in KK2 buffer. Note: Cells submerged in KK2 buffer do not develop past the mound stage. Scale bars (Petri dish) = 250 µm. Scale bar (nitrocellulose membrane) = 1000 µm.

In this study, we showed that neddylation is required for the growth and multicellular development of *D. discoideum*, which complements similar work in other organisms (e.g., parasitic protozoa, fungi, plants, animals, and humans) to better understand the biological roles of neddylation (Figure 8) [23,24,25,26,27]. The expression and amount of free Nedd8 peaked during the early stages of multicellular development, and then decreased dramatically during the mid-to-late stages. Treatment of cells with MLN4924 inhibited cell proliferation and folic acid-mediated chemotaxis during growth, and cAMP-mediated chemotaxis, aggregation, mound formation, and fruiting body formation during development. Combined, these data reveal the importance of neddylation during the *D. discoideum* life cycle, which enhances our understanding of neddylation in eukaryotes.

Our bioinformatic analysis revealed several *D. discoideum* orthologs of human proteins associated with neddylation, suggesting that the neddylation pathway is conserved from *D. discoideum* to metazoans. This included orthologs of human NEDD8, NAE1, and others associated with the E1–E2–E3 cascade. Based on these findings, we examined the ability of the well-established neddylation inhibitor, MLN4924, to inhibit neddylation in *D. discoideum*. We observed increased levels of free Nedd8 in cells treated with MLN4924, suggesting that the chemical inhibited the conjugation of Nedd8 to target proteins.

Treatment of *D. discioideum* with MLN4924 significantly reduced cell proliferation and folic acid-mediated chemotaxis. Consistent with this finding, free Nedd8 was detected in growth-phase cells, and MLN4924 has been reported to inhibit the proliferation of a variety of cancer cell types [75,76,77]. In *D. discoideum*, previous work identified developmental roles for CulA and CulB, with no mention of growth-stage defects [70,73]. While these findings do not exclude the possibility that *culA^−^* or *culB^−^* cells display aberrant phenotypes during growth, they do suggest that there may be cullin-independent roles of neddylation during growth, which is consistent with observations in other organisms [10,11,12]. Growth defects have also been reported in *D. discoideum* cells lacking components of the de-neddylation machinery. In humans, de-neddylation of target proteins occurs via the CSN5 subunit of the CSN [69]. Loss of CSN5 ortholog in *D. discoideum*, *csn5*, severely impairs cell proliferation, suggesting that de-neddylation is important during growth [32]. A previous proteomics approach identified neddylated proteins in *Trypanosoma brucei* (a eukaryotic parasite that emerged prior to plants) and it included a long list of non-cullin proteins involved in a variety of cellular processes such as cell division and metabolism [78]. A number of these non-cullin proteins and their modification by neddylation may be a conserved post-translational modification in *D. discoideum*. Therefore, future work should aim to identify non-cullin neddylated targets in *D. discoideum* to understand the full scope of neddylation-regulated pathways in eukaryotes.

Multicellular development of *D. discoideum* begins with the aggregation of cells in response to the production and secretion of the chemoattractant cAMP. The SCF complex was previously shown to be essential for cAMP-mediated aggregation through its targeting of the cAMP phosphodiesterase RegA for ubiquitination and subsequent degradation [70]. Consistent with this finding, CulA binds RegA in vivo. Cells lacking *culA* accumulate RegA, which subsequently degrades cAMP to prevent chemotaxis and aggregation. In addition, the phenotypes of *culA^−^* cells were linked to an inability of cells to properly activate the cAMP-dependent protein kinase A during multicellular development. These findings mirror the effects of MLN4924 on cAMP-mediated chemotaxis and aggregation observed in this study. They are also consistent with the detection of free Nedd8 during the early stages of *D. discoideum* development as well as multiple reports of MLN4924 inhibiting the migration of cancer cells [79,80,81]. Previous work in *D. discoideum* implicated another ubiquitin-like modification, SUMOylation, in the regulation of chemotaxis. *D. discoideum* mitogen-activated protein kinase kinase (MEK1) is a signaling kinase that regulates chemotaxis and was shown to be a target substrate for SUMOylation and ubiquitination [82]. SUMOylation of MEK1 occurred after chemoattractant stimulation. Similarly, there may be upstream signalling proteins involved in *D. discoideum* chemotaxis that are regulated by neddylation. Collectively, these findings suggest a role for ubiquitin-like post-translational modifications such as neddylation in regulating cell migration in eukaryotes, which is further supported by our observations of reduced folic acid-mediated chemotaxis in *D. discoideum* cells treated with MLN4924.

To gain insight into the pathways that may be affected by MLN4924 treatment in *D. discoideum*, we explored the effect of MLN4924 on the intracellular and extracellular amounts of the cell density-sensing glycoprotein, CmfA [71]. We observed increased amounts of intracellular and extracellular CmfA in cells treated with MLN4924, suggesting that cells may have attempted to compensate for the delayed aggregation by producing and secreting more CmfA. While these findings suggest that treatment with MLN4924 compromises cell–cell signaling during the early stages of development, we could not rule out the possibility that the increased amount of intracellular CmfA was due to impaired proteasome-mediated degradation. Thus, future work aimed at resolving the mechanism disrupted by MLN4924 in *D. discoideum* may consider examining the effect of the chemical on proteasome-mediated degradation. Interestingly, we observed no effect of MLN4924 on the intracellular or extracellular amounts of the calcium-dependent cell adhesion protein CadA, suggesting that MLN4924 may not affect cell-to-cell adhesion mechanisms [72].

Previous work linked the functions of CulA and CulB to morphogenesis and cell differentiation during *D. discoideum* development [70,73]. Spore formation does not occur in the absence of *culA*, and loss of *culB* causes aberrant pre-stalk cell differentiation that ultimately affects fruiting body size. Similarly, when neddylation is inhibited by MLN4924, there is a significant reduction in the percentage of fruiting bodies that develop from multicellular aggregates. It is likely that neddylation inhibition prevents the required assembly of the CulA- and CulB-associated CRLs to facilitate normal differentiation. However, as previously mentioned, there may be other neddylated proteins in *D. discoideum* that are also involved in differentiation and are affected by MLN4924. For example, CarC, a cAMP receptor involved in cell differentiation was previously identified as an interactor of Csn5 [31]. Although it has yet to be shown, it is possible that CarC is neddylated, which could be a contributing factor to the reduced fruiting body formation we observed when cells were treated with MLN4924. Therefore, *D. discoideum* can be used as a model system to better understand how neddylation influences cell fate decisions and differentiation, which can improve our understanding of its role in human development.

## 5. Conclusions

This study demonstrates how *D. discoideum* can be used as a model system to better understand the unicellular and multicellular processes regulated by neddylation. Future work that identifies the targets of neddylation in *D. discoideum* may reveal non-cullin targets of neddylation that could inform research in other organisms to enhance our understanding of neddylation in all eukaryotes. In this paper, we showed a dose-dependent effect of MLN4924 on growth, aggregation, and multicellular development, which suggests the chemical is not toxic to cells and that it acts specifically on Nae1, as in other organisms. While we saw no evidence of cell death during treatment with MLN4924, we cannot rule out the possibility that MLN4924 is toxic at higher concentrations. Finally, this work identifies MLN4924 as an effective inhibitor of neddylation in *D. discoideum* and highlights the potential of *D. discoideum* to serve as a unique biomedical model system for examining the therapeutic potential of MLN4924.

## Data Availability

Not applicable.

## Supplementary Materials

The following are available online at https://www.mdpi.com/2218-273X/11/3/482/s1, Table S1. *D. discoideum* proteins orthologous to human cullin proteins; Table S2. *D. discoideum* proteins orthologous to human proteins involved in the neddylation pathway.

## Abbreviations

The following abbreviations are used in this manuscript:

CadA	calcium-dependent cell adhesion molecule A
CAND1	cullin-associated and neddylation dissociated 1
CmfA	conditioned medium factor
COP9	constitutive photomorphogenesis 9
CRL	cullin–RING ligase
CSN	COP9 signalosome
CUL	cullin
DMSO	dimethyl sulfoxide
NAE1	NEDD8 activating enzyme E1
NEDD8	neural precursor cell expressed, developmentally downregulated protein 8
RBX1	E3 ubiquitin protein ligase
SCF	Skp1–Cullin–F-box
SENP8	sentrin-specific protease 8
Ube1C	ubiquitin-activating enzyme E1C
Ube2M	ubiquitin-conjugating enzyme E2 M
UBE2M	NEDD8-conjugating enzyme Ubc12
Uch1	ubiquitin C-terminal hydrolase 1
Uch2	ubiquitin C-terminal hydrolase 2
UCHL1	ubiquitin carboxyl-terminal hydrolase isozyme L1
UCHL3	ubiquitin carboxyl-terminal hydrolase isozyme L3
UCHL5	ubiquitin carboxyl-terminal hydrolase isozyme L5
